# Repeated Evolution of Fungal Cultivar Specificity in Independently Evolved Ant-Plant-Fungus Symbioses

**DOI:** 10.1371/journal.pone.0068101

**Published:** 2013-07-25

**Authors:** Rumsaïs Blatrix, Sarah Debaud, Alex Salas-Lopez, Céline Born, Laure Benoit, Doyle B. McKey, Christiane Attéké, Champlain Djiéto-Lordon

**Affiliations:** 1 Centre d'Ecologie Fonctionnelle et Evolutive (CEFE), CNRS/CIRAD-Bios/Université Montpellier 2, Montpellier, France; 2 Institut Universitaire de France, Montpellier, France; 3 Département de Biologie, Université des Sciences et Techniques de Masuku (USTM), Franceville, Gabon; 4 Laboratory of Zoology, University of Yaoundé I, Yaoundé, Cameroun; Emory University, United States of America

## Abstract

Some tropical plant species possess hollow structures (domatia) occupied by ants that protect the plant and in some cases also provide it with nutrients. Most plant-ants tend patches of chaetothyrialean fungi within domatia. In a few systems it has been shown that the ants manure the fungal patches and use them as a food source, indicating agricultural practices. However, the identity of these fungi has been investigated only in a few samples. To examine the specificity and constancy of ant-plant-fungus interactions we characterised the content of fungal patches in an extensive sampling of three ant-plant symbioses (*Petalomyrmex phylax*/*Leonardoxa africana* subsp. *africana*, *Aphomomyrmex afer*/*Leonardoxa africana* subsp. *letouzeyi* and *Tetraponera aethiops*/*Barteria fistulosa*) by sequencing the Internal Transcribed Spacers of ribosomal DNA. For each system the content of fungal patches was constant over individuals and populations. Each symbiosis was associated with a specific, dominant, primary fungal taxon, and to a lesser extent, with one or two specific secondary taxa, all of the order Chaetothyriales. A single fungal patch sometimes contained both a primary and a secondary taxon. In one system, two founding queens were found with the primary fungal taxon only, one that was shown in a previous study to be consumed preferentially. Because the different ant-plant symbioses studied have evolved independently, the high specificity and constancy we observed in the composition of the fungal patches have evolved repeatedly. Specificity and constancy also characterize other cases of agriculture by insects.

## Introduction

Ant-plants, or myrmecophytes, are plants that provide symbiotic ants with nesting cavities (specialized hollow structures, called domatia). Ant-plant symbioses involve about 100 plant and 40 ant genera in the tropics and have evolved many times independently [1]. Domatia originate from diverse modified plant structures: twigs, petioles, leaf laminae, stipules, rhizomes or tubers. The symbiotic ants usually obtain a large part of their food from plant products, either directly (extrafloral nectar and food bodies) or indirectly (honeydew produced by hemipterans reared in domatia) [2]. Most associated ant species protect the plant against herbivores, pathogens and competing vegetation [3], [4], [5]. They also often provide their host plant with nutrients [6]. In most cases, each individual plant is occupied by a single colony. In some species, a single colony can occupy several adjacent plants of the same species.

It has become evident that ant-plant symbioses should be considered not as bipartite interactions but as symbiotic communities involving, in many cases, plants, ants, hemipterans, fungi, bacteria and possibly nematodes [7], [8], [9], [10]. This conceptual shift applies to all mutualistic interactions and proves useful for a better understanding of the functioning and evolution of ecosystems [11], [12]. Microorganisms such as fungi have long been noticed within domatia [13], [14], [15], but their identities and roles are just beginning to be understood [10], [16]. They have been detected in most ant-plant symbioses investigated and form a whole set of new species of the order Chaetothyriales (Ascomycota) [10]. They form dense and well delimited mats of hyphae covering a small area on the inner wall of the domatium. Fungal patches occur in limited number, but are present in each domatium of a single plant. The true symbiotic nature of the ant-plant-fungus association was first demonstrated in the African symbiosis between the ant *Petalomyrmex phylax* and the plant *Leonardoxa africana* subsp. *africana* (Fabaceae, subfamily Caesalpinioideae) [17]. Nutrient flux from ants to fungal patches was also demonstrated in this system [18], suggesting a manuring process. Although the role of these fungi remains largely unexplored, ants have been shown to use them as a food source in three ant-plant symbioses, *Pseudomyrmex penetrator*/*Tachigali* sp. (Fabaceae, subfamily Caesalpinioideae), *Petalomyrmex phylax*/*Leonardoxa africana* subsp. *africana* and *Tetraponera aethiops*/*Barteria fistulosa* (Passifloraceae) [16]. Considered together, along with more anecdotal observations, these studies strongly suggest that plant-ants farm these fungi for food. As ant-plant-fungus symbioses have evolved many times independently, they could represent multiple cases of parallel evolution of agriculture.

Fungiculture has been thoroughly investigated in three widely separated insect lineages [19]: fungus-growing ants (tribe Attini), fungus-growing termites (subfamily Macrotermitinae) and ambrosia beetles (Scolytidae, subfamily Scolytinae, including the Platypodinae). In contrast, very few data exist on other potential cases of agriculture conducted by animals. These cases involve damselfish and *Polysiphonia* algae [20], a marine snail and ascomycete fungi [21], gall midges (cecidomyiid flies) and dothideomycete fungi [22], and plant-ants and chaetothyrialean fungi [16], [23], [24]. Investigation of a greater range of agricultural systems is needed to obtain a more comprehensive understanding of the global pattern of the evolution of agriculture by animals, and to compare the features of these diverse and parallel coevolved systems.

In most ant-plant symbioses, the pattern of specificity between ants and plants is well known. However, the extent of specificity of their domatia-inhabiting fungal symbionts has never been assessed. We focussed on three ant-plant symbioses for which evidence strongly suggests that they are new cases of fungiculture by ants [7], [16], [17], [18]: *Petalomyrmex phylax*/*Leonardoxa africana* subsp. *africana*, *Aphomomyrmex afer*/*Leonardoxa africana* subsp. *letouzeyi* and *Tetraponera aethiops*/*Barteria fistulosa*. We aimed at characterising (i) the fungal community within domatia over a large number of samples in order to test for ant-plant-fungus specificity and (ii) the geographic pattern of variation in the occurrence of the specific fungal taxa in order to assess the degree of interdependency among the associated species. Sexual structures of domatia fungi have never been observed in fungal patches tended by ants and identification of species from hyphae is not possible. We thus used a stepwise DNA barcode approach, using polymerase chain reaction (PCR) with universal and then specific primers, to characterise the identity and distribution of the fungal partners associated with each of these ant-plant symbioses.

## Methods

The symbiosis between the ant *Petalomyrmex phylax* and the plant *Leonardoxa africana* subsp. *africana* is obligatory, highly specific and endemic to the coastal rain forest of southern Cameroon [25]. A total of 98 fungal patches were sampled from 80 individual trees distributed along an 85-km transect of coastal forest, covering almost half the distribution area of this symbiosis. For 11 trees we sampled several domatia (two to five).

The ant-plant *Leonardoxa africana* subsp. *letouzeyi* can be occupied by non-specific ants at the sapling stage, but when trees are mature, the obligatory plant-ant *Aphomomyrmex afer* is by far the most common inhabitant [25], [26]. This symbiosis is restricted to the lowland rain forests near the Bight of Biafra, across the boundary between Cameroon and Nigeria [25]. A single fungal patch was sampled from each of 17 individual trees occupied by *A. afer*, in a single site, around Iriba Inene camp in Korup National Park, Cameroon.

The ant-plant *Barteria fistulosa*, whose lateral branches are hollow throughout their length, and its ant symbiont *Tetraponera aethiops* are widely distributed over the whole Lower Guinea - Congo basin forest block [27]. They are considered highly dependent on each other because the ant has never been found nesting outside a *Barteria*, and unoccupied plants do not grow well [14]. However, *B. fistulosa* can also be found in association with the ant *Tetraponera latifrons*, and both *Tetraponera* spp. can also colonise the related and morphologically similar plant *Barteria dewevrei* [27], [28]. A total of 440 fungal patches were collected in Cameroon and Gabon from 411 individual trees of *B. fistulosa* occupied by *T. aethiops*. Samples were collected over an area of nearly 100 000 km^2^. For 13 trees we sampled several domatia (two to five).

The following authorities provided research permits and permitted sample collection: Ministry of Scientific Research and Innovation of the Republic of Cameroon, the conservator of Korup National Park (Cameroon), Université des Sciences et Techniques de Masuku (Gabon), Ministère de l'Education Nationale, de l'Enseignement Supérieur et Technique et de la Formation Professionnelle de la Recherche Scientifique chargé de la Culture, de la Jeunesse et des Sports (Gabon).

Fungal samples were either dried under silica gel or stored in extraction buffer immediately upon collection in the field. DNA was extracted using either the modified CTAB method described in [10] or the REDExtract-N-Amp Plant PCR Kit (Sigma–Aldrich, St. Louis, USA). Fungus identities were assessed by sequencing approximately 600 bp of the Internal Transcribed Spacer (ITS) of the nuclear ribosome, which comprises ITS1, 5.8S and ITS2, and is considered to be the best universal DNA barcode marker for fungi [29].

The first step for each biological system studied was to sequence ITS using fungal universal primers ITS1f [30] and ITS4 [31] for all fungal patches from the systems *Petalomyrmex/Leonardoxa* (98 samples) and *Aphomomyrmex/Leonardoxa* (17 samples), and for 78 fungal patches (out of 440) from the system *Tetraponera/Barteria*. This step allowed identifying the fungal taxa associated with each symbiosis. For the *Petalomyrmex/Leonardoxa* and *Aphomomyrmex/Leonardoxa* systems we performed molecular cloning respectively on nine and one first-step PCR products for which the sequence could not be read. This first step indicated that two specific fungal taxa occurred in each of the two systems *Petalomyrmex/Leonardoxa* and *Tetraponera/Barteria* and one specific taxon in the system *Aphomomyrmex/Leonardoxa*. However, this method did not allow determining whether two fungal taxa could co-occur in a single patch (except for the few samples on which we performed molecular cloning). We thus applied a second step, which involved only the two systems *Petalomyrmex/Leonardoxa* and *Tetraponera/Barteria*. This step consisted in testing for the presence of each specific fungal taxon in each fungal patch. For this, we designed primers specific to each of the Chaetothyriales taxa detected in the first step (primer sequences are given in Table 1).

**Table 1:** Sequences of primers developed in this study to amplify specifically the ITS region of fungal Molecular Operational Taxonomic Units (MOTU) detected in two focal ant-plant symbioses.

MOTU targeted	Name of primer	Sequence 5′ - 3′	associated ant-plant symbiosis
La1	its1La1	GAGTGAGGGTCTCTGTGCCC	*Petalomyrmex/Leonardoxa*
	its4La1	TACAACTCGGACCCCAAGGGGC	
La2	its1La2	GTTAGGGTTCCTCTCACGGG	*Petalomyrmex/Leonardoxa*
	its4La2	AAATTACAACTCGGGCCGTG	
Y1	its1Y1	GGCTGCCGGGGGGTTCTATT	*Tetraponera/Barteria*
	its4Y1	GTCAACCTTAGATAAAACTA	
Y9	ITS1f is used as forward primer		*Tetraponera/Barteria*
	its4Y9	TCAACCTTTAGATATAAGA	

To control whether the specific primers amplified the taxa they were respectively designed for, we sequenced all PCR products for the *Petalomyrmex/Leonardoxa* system. For the *Tetraponera/Barteria* system, 40 PCR products obtained with the two specific ITS primer pairs yielded sequences of the targeted species, confirming the high specificity of the primers in this system. As a consequence, 362 samples out of 440 were simply screened for the presence of each specific fungal taxon through success or failure of amplification with the specific primers (but no sequencing of PCR product).

Amplifications were performed in a 25 µl solution containing 1× PCR mix (multiplex kit, Qiagen, Venlo, Netherlands), 0.5 µM of each primer and 1 µl of DNA template. They took place in a thermal cycler programmed for an initial denaturation step of 15 min at 95°C, followed by 35 cycles of 60 s at 94°C, 60 s at 53°C and 60 s at 72°C, and a final elongation step of 20 min at 60°C. Molecular cloning of PCR product was performed using the kit pGEM-T easy vector (Promega, Madison, USA) and following the manufacturer's instructions.

ITS sequences were first searched for relatives using the Basic Local Alignment Search Tool in GenBank (http://blast.ncbi.nlm.nih.gov/Blast.cgi). This allowed detecting which sequences belonged to species of the order Chaetothyriales. Sequences of Chaetothyriales were then classified into haplotypes. One sequence for each haplotype was deposited in GenBank. All haplotypes from the three systems were aligned with Muscle [32] and a maximum likelihood tree was constructed using PhyML [33] in order to guide our choice for delimitation of Molecular Operational Taxonomic Units (MOTUs, sensus [34]). In addition, we used the conservative cut-off value of 95% ITS sequence similarity for delimitation of MOTUs. Although higher cut-off values have been proposed in previous studies considering ITS as fungal barcodes [29], [35], we prefer to use a conservative value because intra-specific sequence variation can vary across taxonomic groups and Chaetothyriales fungi are poorly known in this respect. Thus, the splitting into taxonomic units that we propose in this paper is likely to remain valid in the future.

## Results

**Figure 1:**
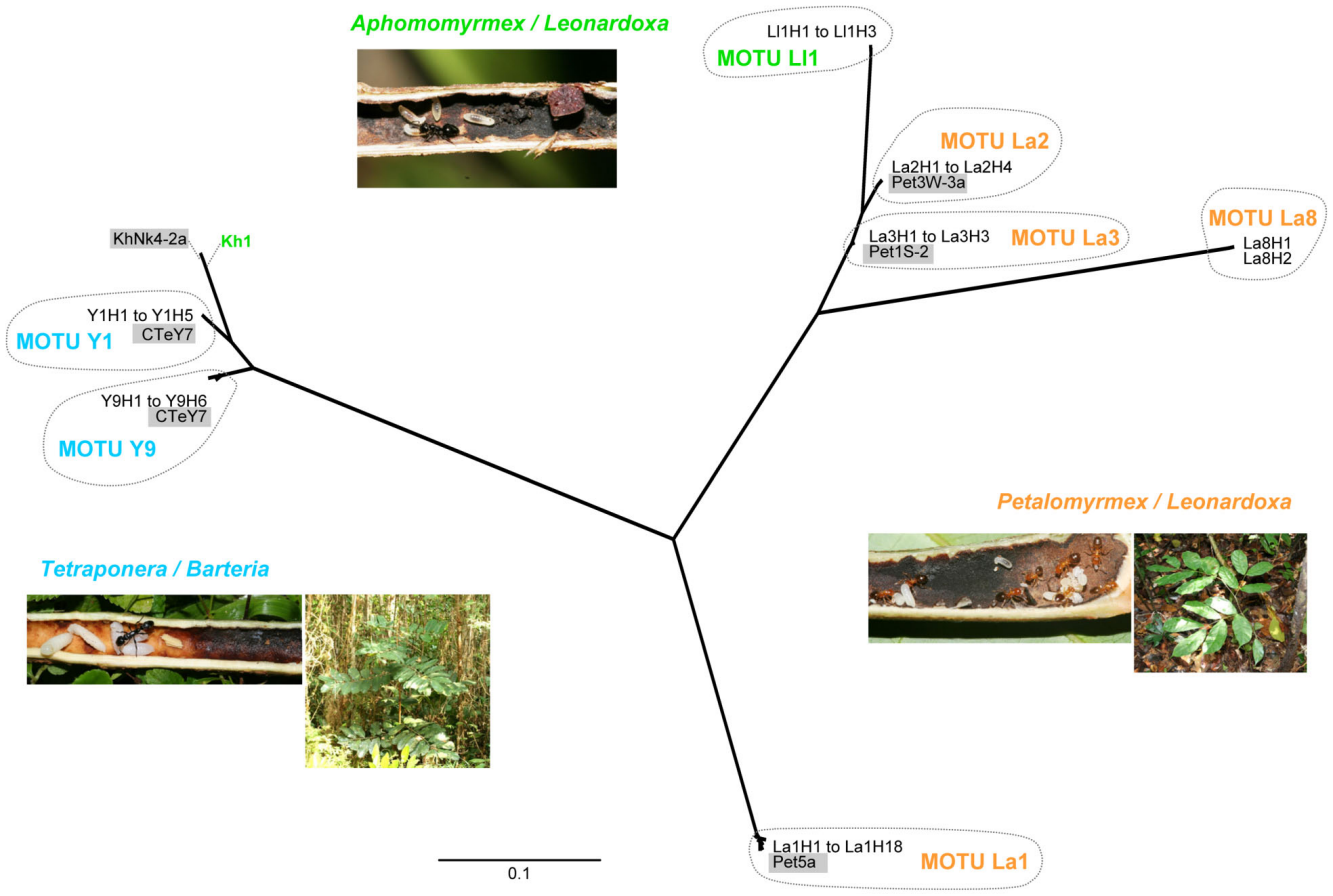
Maximum likelihood tree of ITS fungal haplotypes from three ant-plant-fungus symbioses. A total of 42 haplotypes (based on sequences of 647 aligned nucleotides) of Chaetothyriales were detected in fungal patches of the following ant-plant symbioses: *Petalomyrmex phylax/Leonardoxa africana* subsp. *africana* (MOTUs in orange), *Aphomomyrmex afer/Leonardoxa* subsp. *letouzeyi* (MOTU in green) and *Tetraponera aethiops/Barteria fistulosa* (MOTUs in blue). Note that haplotype Kh1 is labelled in green because it was detected in the system *Aphomomyrmex*/*Leonardoxa*, although it is phylogenetically most closely related to MOTU Y1. The position of each MOTU on the tree is indicated by the intersection of the branches and the dotted lines. Branch tip labels highlighted in grey correspond to fungal strains obtained in previous studies following a culturing approach. For each symbiosis, the image on the left displays a domatium cut longitudinally to expose ants and a fungal patch (dark area on the inner surface). MOTU: Molecular Operational Taxonomic Unit.

**Figure 2:**
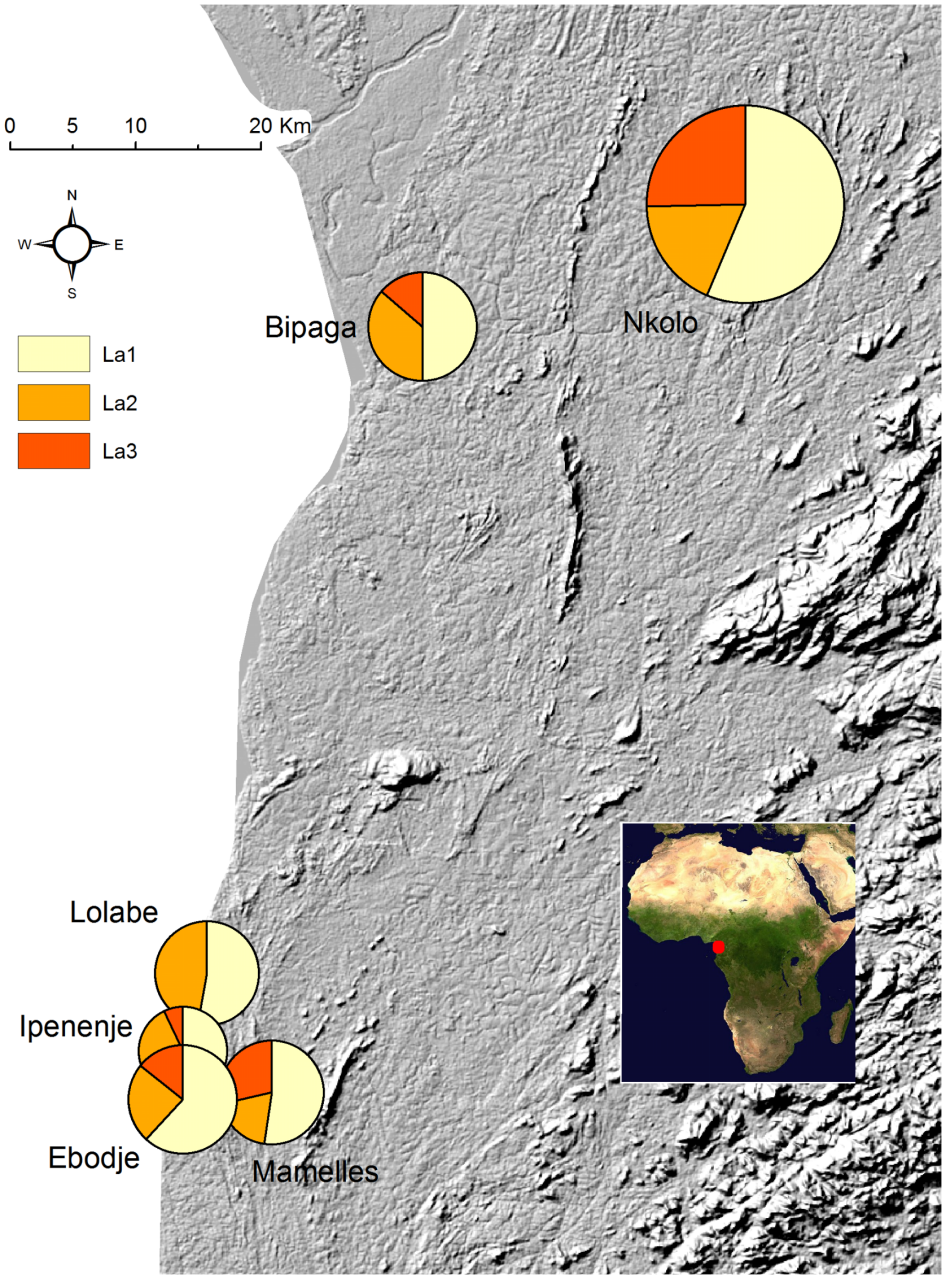
Spatial distribution of Chaetothyriales MOTUs of the *Petalomyrmex phylax/Leonardoxa africana* subsp. *africana* system. Sectors represent the proportion of each Molecular Operational Taxonomic Unit in each sampling site. MOTUs were detected using universal and/or specific ITS primers. Size of pie charts is proportional to sample size (i.e., the number of fungal samples for which at least one Chaetothyriales MOTU was detected).

**Figure 3:**
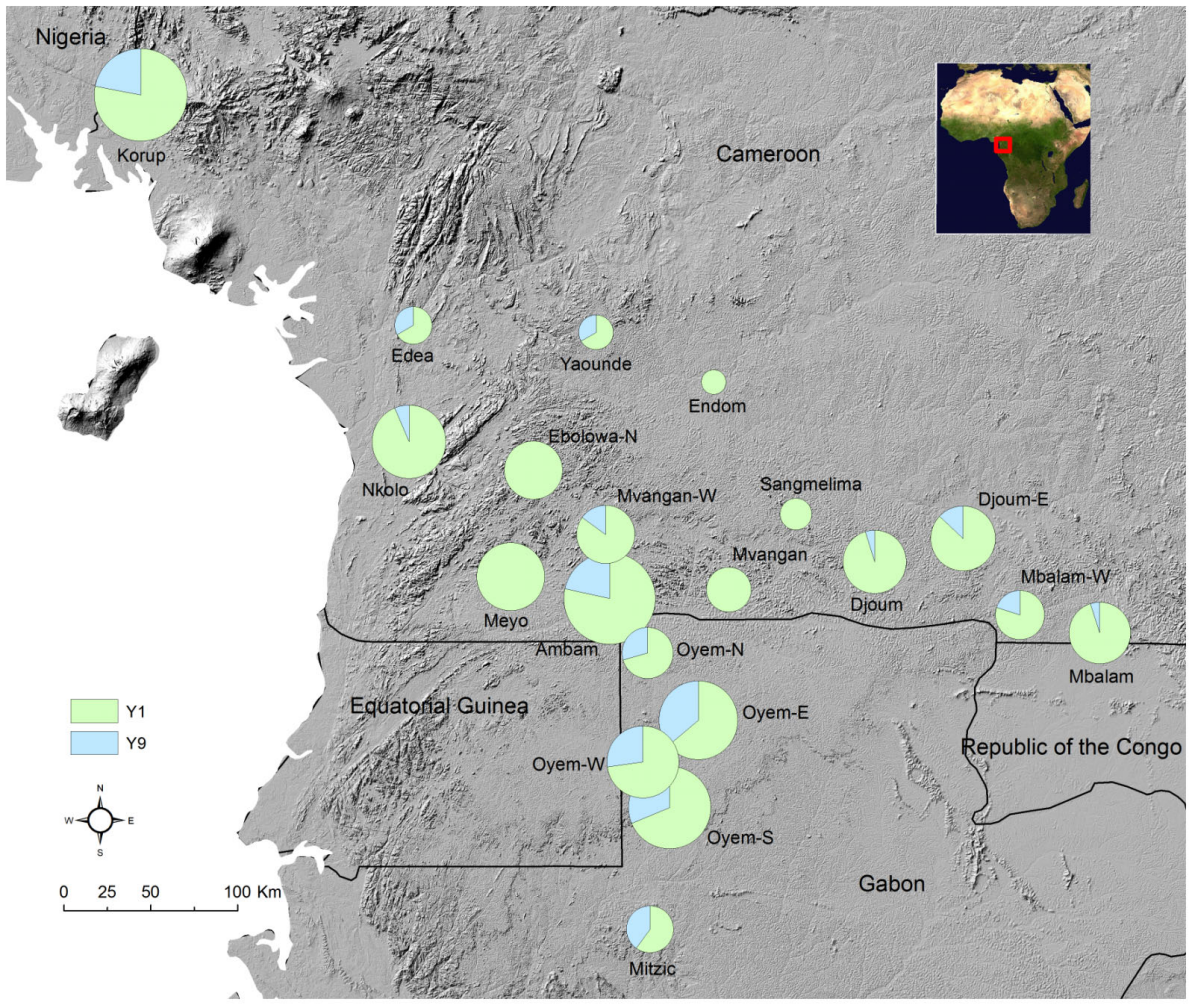
Spatial distribution of Chaetothyriales MOTUs of the *Tetraponera aethiops/Barteria fistulosa* system. Sectors represent the proportion of each Molecular Operational Taxonomic Unit in each sampling site. MOTUs were detected using universal and/or specific ITS primers. When specific ITS primers were used, PCR products were not always sequenced. Size of pie charts is proportional to sample size (i.e., the number of fungal samples for which at least one Chaetothyriales MOTU was detected).

Out of a total of 311 ITS sequences, 305 were sequences of Chaetothyriales for the systems *Petalomyrmex/Leonardoxa* (208 sequences), *Aphomomyrmex/Leonardoxa* (10 sequences) and *Tetraponera/Barteria* (87 sequences), and only six sequences were of a different order. According to Blast results, these last six sequences most likely belonged to *Candida* (Ascomycota, Saccharomycetales), *Cryptococcus* (Basidiomycota, Tremellales), *Neurospora* (Ascomycota, Sordariales), *Fusarium* (Ascomycota, Hypocreales) and a Capnodiales (Ascomycota). From the Chaetothyriales sequences, we detected a total of 42 haplotypes (GenBank accession numbers KC951221 to KC951262) that were grouped into eight likely MOTUs (Fig. 1). For 7% of the sequences we could not determine the haplotype because of low sequence quality at determinant positions. We found four, two and two Chaetothyriales MOTUs in the *Petalomyrmex/Leonardoxa*, *Aphomomyrmex/Leonardoxa* and *Tetraponera/Barteria* systems respectively. Each symbiosis had its own set of MOTUs. Within each MOTU, haplotypes had more than 98.6% similarity in ITS sequence. The two most closely related MOTUs, La2 and La3, had ITS sequences similar at 95%. For the two systems with multiple sampling sites (*Petalomyrmex/Leonardoxa* and *Tetraponera/Barteria*), distribution of MOTUs did not seem to show spatial structure (Fig. 2 and Fig. 3).

For the *Petalomyrmex/Leonardoxa* system, samples for which a readable sequence was obtained using universal primers yielded La1 and La2 in 96% and 4% of the cases respectively (Table 2). We designed specific ITS primers for La1 and La2 (Table 1). In this system, all PCR products obtained with specific primers were sequenced. Sequences obtained with primers specific to La1 always yielded La1, whereas sequences obtained with primers specific to La2 yielded either La2 or La3. Molecular cloning of PCR products allowed detection of up to five haplotypes of a single MOTU in a single fungal patch. The number of haplotypes detected only when PCR products were cloned was 14 (out of 18), two (out of four) and zero (out of three) for La1, La2 and La3 respectively, showing that diversity within MOTUs is underestimated without molecular cloning. However, cloning revealed only one additional MOTU (La8). When we combine the results from all methods (PCR with universal or specific primers and molecular cloning of PCR products obtained with universal primers) La1, La2, La3 and La8 were detected in 97%, 47%, 32% and 2% of the samples respectively (Table 2). In 76% of the samples we detected both La1 and either La2 or La3. We cannot rule out the possibility that La2 and La3 co-occur in the same samples because we did not test diagnostic primer pairs. In 21% of the samples we detected La1 only. In 3% of the samples we detected either La2 or La3 only. In half of the plant individuals for which we sampled several fungal patches (one per domatium) we found exactly the same MOTUs in all patches from the same individual. In the other half we found patches with La1 only and patches with La1 and either La2 or La3 in the same individual.

**Table 2:** Number of fungal samples in which the different MOTUs were detected using sequencing of the ITS region (ITS1, 5.8S, ITS2) of ribosomal DNA.

	Universal primers^a^					All methods^b^					
*Petalomyrmex/Leonardoxa*	La1	La2	NS^c^		Total	La1	La2	La3	La8	others^d^	Total
	80	3	15		98	95	46	31	2	1	98
*Aphomomyrex/Leonardoxa*	Ll1	others^d^	NS^c^		Total	Ll1	Kh1	others^d^	NS^c^		Total
	8	2	7		17	9	1	3	6		17
*Tetraponera/Barteria*	Y1	Y9	others^d^	NS^c^	Total	Y1	Y9	others^d^	NS^c^		Total
	31	12	2	33	78	369	91	2	53		440

a PCR was performed directly on the fungal patch using fungal universal primers ITS1f and ITS4, and thus only one species per sample can be detected.

b species were detected using either universal primers, molecular cloning of PCR product or species-specific primers, so that several species per sample can be detected.

c either no amplification, or the sequence was not readable.

d sequences that do not belong to Chaetothyriales (likely contaminants or non-symbiotic competitors).

In the *Aphomomyrmex/Leonardoxa* system, for 41% of the samples we did not obtain a readable sequence with universal primers. The other samples yielded Ll1 in eight out of 10 (80%) of the cases (Table 2). In the other two cases, the sequences revealed fungi that did not belong to the Chaetothyriales and that were likely contaminants or non-symbiotic competitors (*Candida*, *Fusarium*). For the one sample on which molecular cloning was performed, we detected Ll1, Kh1 and a Capnodiales. Kh1 is similar to the Chaetothyriales strain KhNk4-2a that has previously been isolated from the symbiosis between the African plant *Keetia hispida* (Rubiaceae) and ants of the genus *Crematogaster* [10], which can be found in the forest where we sampled the *Aphomomyrmex/Leonardoxa* system.

For the *Tetraponera/Barteria* system, samples for which a readable sequence was obtained using universal primers yielded Y1 and Y9 in 69% and 27% of the cases respectively (Table 2). In the other cases (two out of 78), the sequences revealed fungi that did not belong to the Chaetothyriales and that were likely contaminants or non-symbiotic competitors (*Neurospora, Cryptoccocus*). Specific primers were designed for both Y1 and Y9 (Table 1). When we combine the results from all methods (PCR with universal or specific primers), Y1 and Y9 were detected in 84% and 21% of the samples respectively (Table 2). In 17% of the samples we detected both Y1 and Y9. In 67% of the samples we detected Y1 only. In 4% of the samples we detected Y9 only. In the other samples (12%) neither Y1 nor Y9 were detected. This high proportion of amplification failure is likely due to low quality of DNA extraction. Moreover, we did not repeat unsuccessful PCR for the 362 samples (out of 440) that were screened with specific primers and for which PCR product was not sequenced. In several plant individuals for which we sampled several fungal patches we found differences in MOTU composition among patches of a single individual. A young *B. fistulosa* individual (BF365) contained four founding queens, each in a separate domatium. A fungal patch was associated with each of these queens. For two patches amplifications failed. For the two others amplification was successful with Y1-specific primers and failed with Y9-specific primers.

Details on each individual sample of fungal patch, including collection information and detected MOTUs, are available in Table S1.

## Discussion

The DNA barcode approach that we used on direct extracts of DNA fungal patches from three ant-plant symbioses detected mostly taxa belonging to the Chaetothyriales. In fact, fewer than 2% of the sequences were from other orders. From microscopic observation and culturing of fungal patches in previous studies [10], [17] we know that many different fungi are present as spores or fragments of hyphae but do not grow in the natural conditions of domatia occupied by mutualist ants. The few sequences of non-Chaetothyriales taxa most likely represent such fungi that may have reached the domatia opportunistically. Previous studies showed that many ant-plant symbioses are associated with Chaetothyriales [7], [10], [36] and the present results confirm for three symbioses that this type of association is consistent over a large sampling.

Although intra-specific ITS sequence variability varies across taxa, it averages 2.5% in fungi, and more specifically, less than 2% in Ascomycota [29], [35]. Applying mean cut-off values for species delimitation in poorly known groups, such as Chaetothyriales, is likely to bring erroneous conclusions. However, classification of the sequences from this study (42 haplotypes of Chaetothyriales) into MOTUs was rather straightforward through visual inspection of the Maximum Likelihood phylogenetic tree (Fig. 1). Moreover, sequence variability was less than 2% within and more than 5% between defined MOTUs. Although delimited MOTUs are likely to correspond to species, we are reluctant to use this term before more molecular data are available.

In our study models successful direct amplification of fungal patches with universal primers yielded one main MOTU in each system investigated. Amplification with primers specific for this primary MOTU revealed that it was also present in most samples in which it was not detected with universal primers. In the *Petalomyrmex/Leonardoxa* system two other secondary MOTUs were commonly detected using specific primers but only very rarely when using universal primers. This suggests that the primary MOTU is quantitatively the most abundant in fungal patches but that another MOTU occurs alongside. Moreover, the secondary MOTUs were very rarely detected alone, without the primary one. We did not note any particularity that was shared by the samples in which only the secondary MOTUs were detected. A likely explanation for these cases is amplification failure of the primary MOTU due to poor quality of DNA extracts for these samples. In the *Tetraponera/Barteria* system, we detected only one secondary MOTU. In this system, both primary and secondary MOTUs were detected using universal primers and the proportion of samples that had only one of them (detected with specific primers) was higher than in the *Petalomyrmex/Leonardoxa* system. The nature of the relationship between ants and the secondary MOTUs might differ between the two systems, as the pattern of occurrence appears different. The fungi associated with the three ant-plant symbioses are different between the symbioses, even when they occur in sympatry. For instance, in one sampling site (Nkolo, Cameroon) we collected specimens of the two symbioses, *Petalomyrmex/Leonardoxa* and *Tetraponera/Barteria*, that were only a few tens of meters apart, and still they did not share fungal MOTUs. Clearly, MOTUs are consistently the same between individuals within each study model. For two of these symbioses the sampling covered a substantial part of the distribution, and showed no geographic variation in the identity of fungal symbionts. Altogether, this information shows that Chaetothyriales symbionts associated with our focal ant-plant symbioses are specific to the symbiosis. However, it seems possible that some Chaetothyriales move more freely among systems. For instance, in the *Aphomomyrmex/Leonardoxa* system, in addition to the primary MOTU, we obtained one sequence (through molecular cloning of PCR product) that was very similar to the sequence of a strain isolated and cultured previously from the symbiosis between the small tree *Keetia hispida* (Rubiaceae) and an ant of the genus *Crematogaster* [10], found in the same forest. This may be explained either by dispersal of the fungus or by contamination between samples during lab processing. The extent of sharing of fungal taxa and the presence of these taxa in the environment still remain to be investigated.

The *Petalomyrmex/Leonardoxa* and *Tetraponera/Barteria* systems involve ants and plants that belong to different subfamilies and families respectively. These symbioses are phylogenetically independent. The similarity in their global pattern of specificity with the fungal symbionts thus reflects a repeated pattern in evolution. These two ant-plant symbioses are highly specialised. Further work should describe the pattern of specificity of fungal symbionts in less specialised systems, to test whether cultivar specificity is correlated with ant-plant specialisation. Patterns of specificity are known to vary in other cases of agriculture by insects. In Attine ants, for instance, species in the genera *Acromyrmex* and *Atta* (higher Attines, or leaf-cutting ants) share a unique species of fungal symbiont that they grow in pure culture [37], [38]. In contrast, in lower Attines a single species can use various fungal symbionts, because each species exchanges cultivars horizontally with neighbouring colonies of different ant species [39], [40]. In fungus-growing termites, although most species seem to be associated with a single fungal strain, some can associate with different species of the symbiotic *Termitomyces* fungi [41].

Our study revealed that the two different fungal symbionts that are associated with a single ant-plant symbiosis frequently co-occur in each ant colony. In contrast, in higher Attine ants and Macrotermitinae, each colony seems to rely on the monoculture of a single fungal species [39], [40], [42], [43], even in ant species that can use various fungi. Plant-ants may thus have a mode of agriculture more similar to that of ambrosia beetles, whose fungal gardens are composed of several species of fungi and bacteria [44], [45]. Interestingly, these gardens contain a primary, dominant, fungal strain, along with secondary strains [46], a pattern very similar to that we describe in ant-plant-fungi symbioses. The nature of the interaction between ambrosia beetles and their secondary symbionts is not always understood and secondary symbionts, along with bacteria, may play roles in the agricultural process. Even in higher Attine ants, in which the agricultural process was first considered to involve a limited number of coevolving symbionts [47], a whole community of recruited symbionts are now suspected to play roles [48], [49], [50].

Molecular cloning allowed the detection of several haplotypes of the same MOTU in a single fungal patch. These haplotypes could correspond to different ITS copies from a single individual, but rRNA gene clusters and their spacers are usually homogenised by the process of gene conversion. An alternative explanation is the occurrence of several individuals of the same MOTU in a single fungal patch. In addition, sequencing several fungal patches from a single ant colony (corresponding to one plant individual) revealed variation in their composition in some cases. For instance, one patch could contain the primary MOTU and another one the secondary MOTU. This shows that the ants do not grow a single cultivar that is propagated clonally, but instead combine different individuals and strains. A strict clonal propagation of cultivars is very unlikely to occur in insect agriculture. It has long been thought that this was the mode of propagation of the symbiont in higher Attine ants because each founding queen starts its new fungal garden from hyphae taken from its mother colony before the nuptial flight, and each colony grows a single strain [51], [52]. However, it is now clear that recombination and horizontal transfers occur regularly [39], [40], [53], with monoculture being maintained owing to strain incompatibility [37], [43].

As soon as they produce domatia, saplings of *Barteria fistulosa* are colonised by several founding queens of *Tetraponera*, each of which barricades itself in a single separate domatium by using debris to plug its entrance hole (claustral foundation). When one founding colony has reached a critical size, the workers begin to patrol outside of the domatium and kill all the other founding colonies present on the tree [54]. In the course of this study we collected from a single sapling four founding queens with brood that were still locked in their respective domatia. Each of the four domatia contained a fungal patch, and amplification was successful for two of them. Both yielded Y1 but not Y9. As we never found fungal patches in unoccupied domatia, this suggests that the fungal cultivar is brought by the founding queen either from her mother colony or passively from the environment. In the last case, founding queens would probably also introduce non-symbiotic fungi into domatia and the specificity of the association would likely be achieved through growth on an ant-specific medium that selectively favours particular Chaetothyriales fungi. Although the number of samples we were able to obtain from foundations was very low, the occurrence of the sole strain Y1 suggests that primary and secondary fungal symbionts may have different propagation dynamics. Interestingly, a previous experiment showed that *T. aethiops* ants feed preferentially on Y1, the primary symbiont, rather than on Y9 [16]. Whether Y9 represents a non-preferred symbiont, or a parasite of the system that queens avoid when founding a new colony, deserves further investigation. Ambrosia beetles of the tribe Xyleborini also treat primary and secondary fungal symbionts differently. In most cases, pseudo-vertical transmission by the beetles concerns only the primary symbiont, which is also the one that provides the highest nutritional benefits [19], [46], [55]. For a better understanding of agricultural practices in ant-plant-fungus interactions, further work should link the way ants manage primary and secondary fungal symbionts with the nature of their relationships.

Patterns and processes in agriculture by insects have been thoroughly investigated only in a very limited number of groups: Attine ants, Macrotermitinae and ambrosia beetles. Moreover, each of the first two groups arose from a single evolutionary event, followed by radiation. We thus need to study a much broader range of evolutionarily independent cases of agriculture to understand which mechanisms led repeatedly to successful exploitation of crops. In this context, ant-plant-fungus symbioses are promising models because they are diverse and have evolved many times independently. The very widespread occurrence of chaetothyrialean fungi-ant-plant symbioses suggests they may have a common evolutionary antecedent, such as looser associations of these fungi with non-symbiotic ants. The results presented in this study reveal consistency in patterns of species association. Further comparative analysis of agricultural processes in these symbioses will broaden our understanding of the evolution of agricultural practices by insects.

## Supporting Information

Table S1 Detailed information on each individual sample of fungal patch used in this study: code used in the laboratory where genetic analyses were performed (CEFE), species of associated plant and ant, country where the sample was collected, name of the closest village or town, date of collection, geographical coordinates (WGS84, decimal degrees), name of collector, identity of the Molecular Operational Taxonomic Unit and the corresponding haplotype detected using universal or specific primers or molecular cloning.
(XLSX)
